# Prevalence of *Plasmodium falciparum *infection in asymptomatic rural Gabonese populations

**DOI:** 10.1186/1475-2875-10-33

**Published:** 2011-02-09

**Authors:** Dieudonné Nkoghe, Jean-Paul Akue, Jean-Paul Gonzalez, Eric M Leroy

**Affiliations:** 1 Centre International de Recherches Médicales de Franceville (CIRMF), Franceville, Gabon; 2 Ministry of Health, Libreville, Gabon; 3 Department of Immunodeficiency and Infectious Diseases, University of Liege, Belgium; 4 UMR 190 Emergences des pathologies virales, Université Aix- Marseille II, Institut de Recherches pour le Développement, Marseille, France

## Abstract

**Background:**

Malaria may be perennial or epidemic in sub-Saharan Africa, and its transmission may be stable or unstable, depending on the region. The prevalence of asymptomatic *Plasmodium falciparum *carriage is poorly documented in Gabon. A large survey of *P. falciparum *infection was conducted in asymptomatic individuals living in rural Gabon.

**Methods:**

Two hundred and twenty-two villages were randomly selected in the nine administrative regions. With the participants' informed consent, blood samples were collected for thick and thin blood film examination after 20% Giemsa staining. Prevalence rates were calculated per village, per region and per ecosystem, and nationwide. Demographic risk factors were identified with STATA software version 9.0. Significance was assumed at p < 0.05.

**Results and discussion:**

The prevalence of *P. falciparum *in adults was 6.2% (269/4342) nationwide, with a maximum of 37.2% in one village; a linear decrease was observed with increasing age (p = 0.045). Only 5% of the 399 children from forest areas tested positive. The prevalence was significantly higher in forest areas (7%) than in savannah (4%) and lakeland (2.5%). Within the forest region, the prevalence was significantly higher in forest grassland (10.9%) than in the mountain forest (3.5%), interior forest (6.8%) and north-eastern forest (4.5%).

**Conclusion:**

*Plasmodium falciparum *carriage remains high among adults in rural Gabon. Control measures must be adapted to the region and ecosystem. Routine treatment of asymptomatic individuals should be considered.

## Background

Malaria remains the leading cause of mortality and morbidity in sub-Saharan Africa, with 208 million cases and 863,000 deaths reported in 2008 [1]. Depending on the region, malaria may be perennial or epidemic, and transmission stable or unstable. Children are particularly at risk of severe malaria in endemic areas. Protective immunity is acquired throughout life, and reinfection is thus more likely to be asymptomatic or uncomplicated in adulthood. Patients and asymptomatic carriers constitute the parasite reservoir. Most Gabonese epidemiological studies have focused on children. About 40% of children presenting to a hospital with fever between 2000 and 2002 had a *Plasmodium falciparum-*positive blood film, while a prevalence of 65% was found among healthy schoolchildren in the north in the 1980s [2,3]. Gabon has been classified as a highly endemic country with perennial transmission. Since the emergence of multidrug resistance in *P. falciparum*, new control strategies have been implemented by the Gabonese Ministry of Health in 2005 (prompt and effective treatment of clinical malaria cases with artemisinin-based combination therapy, sulphadoxine-pyrimethamine for intermittent preventive treatment of pregnant women, and insecticide-treated bed nets) and the prevalence of malaria among febrile children living in the capital fell between 2000 and 2008 [4].

Here, the distribution of *P. falciparum *carriage in rural Gabonese populations, based on a large survey of asymptomatic individuals, is reported.

## Methods

The survey focused on rural populations of Gabon, nearly 80% of which is covered by rain forest. The *forest *extends from west to east, from the coastal basin, with the grassland, the mountains, the interior and north-eastern forests. The south and southeast contain isolated areas of *savannah *and steppe. A coastal and continental marine ecosystem (*lakeland*) is located around the mouth of the river Ogooué. The annual average temperature is 26°C and humidity exceeds 80%. Two rainy seasons alternate with two dry seasons. The rainy seasons last a total of about 140 days a year.

A stratified random sampling method was used. The stratification was based on the nine provinces. The required sample size was calculated on the basis of an estimated prevalence of 5 to 10% (using n = ε ^2 ^[p (1-p)]/e ^2^; with ε = 1.96 (alpha risk = 5%), e (precision) = 2% and p = expected prevalence; with n varying from 188 to 864). In each province, between 10 and 40 villages were randomly selected. The villages were geolocated. All healthy adult volunteers (defined as individuals with no complaints requiring medical intervention in the past four weeks) residing in the village for more than one year, and who accepted to give a blood sample, were included in the study.

A supplementary field study was conducted in children living in areas with high *P. falciparum *prevalence rates in adults. Six villages were selected at random, and the children were sampled at their schools with their parents' and teachers' consent. Blood samples were collected from healthy individuals, with their informed consent, into Vacutainers containing EDTA (VWR International, France). Thick and thin blood films were stained with 20% Giemsa and examined for malaria parasites by two experienced microscopists. A sample was considered negative if no parasites were seen in 100 oil-immersion fields (x100 magnification). In case of discrepancies, the slides were read again by the two technicians. Together, they agreed for the final results. Prevalence rates were estimated nationwide and for the different ecosystems. The chi2 test and Fisher's exact test were used to identify risk factors for *P. falciparum *carriage. Univariate crude conditional maximum likelihood estimates of odds ratios (OR) and exact 95% confidence intervals (CI) were determined for each potential risk factor, using STATA software version 9.0 (Stata Corporation, College Station, USA). Significance was assumed at p < 0.05. This study was approved by the Gabonese Ministry of Health

## Results

Between July 2005 and May 2008, 4,342 adults aged from 15 to 85 years were sampled in 220 randomly selected villages in the nine provinces, representing 10.3% of all villages in Gabon. The M:F sex ratio was 0.89. In addition, 399 children younger than 15 years in six villages of Ogooue Ivindo province, north-eastern Gabon, were also sampled; the sex ratio was 1.27, and 39% of the children were younger than nine years.

**Figure 1:**
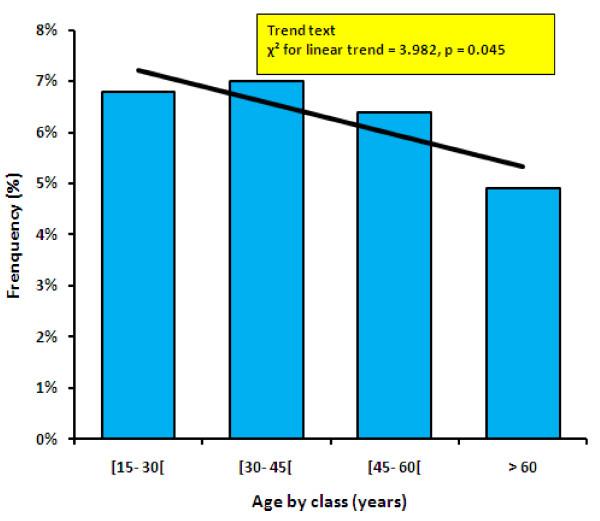
Prevalence of *Plasmodium falciparum *according to age, in the adults group

The prevalence of *P. falciparum *in the adult population was 6.2% overall (95%CI: 5.5- 7), reaching 37.2% in one village (424 inhabitants, 43 tested). The highest prevalence was seen in Estuaire province (14.2%) and the lowest in Ogooue Maritime province (0.5%) (Table 1). The prevalence of *P. falciparum *in the children was 5% overall (95%CI: 3.2- 7.8), and ranged from 1.7% to 8.7% in the different villages. Prevalence did not correlate with gender or age in the child population. However, in adults, a linear decrease (χ^2 ^for linear trend = 3.982, p = 0.045) was observed with increasing age (Figure 1). The prevalence rate was significantly higher (p < 0.0002) in the forest region (7%) than in the savannah (4%) and lakeland (2.5%). No difference (p = 0.2) was observed between savannah and lakeland. Within the forest ecosystem, the prevalence was significantly higher (p < 0.0001) in the grassland (10.9%) than in the mountain forest (3.5%), interior forest (6.8%), and north-eastern forest (4.5%) (Table 2). Finally, the prevalence rate was significantly higher in the rainy season (p < 0.0001).

**Table 1:** Prevalence of *Plasmodium falciparum *carriage, based on thick blood films, among adults, according to the administrative region

Province	Sampling period	Villages		Population		+	%	95%CI
					
		Total	surveyed	Total	surveyed			
Estuaire	July 2005	100	30	53,459	309	44	14.2	10.6- 18.8
Woleu Ntem	April 2006	630	34	95,910	943	87	9.2	7.5- 11.3
Haut Ogooue	April 2007	171	18	50,196	364	29	8	5.5- 11.4
Moyen Ogooue	January 2006	226	31	31,089	611	44	7.2	5.3- 9.6
Nyanga	January 2007	113	16	20,544	412	28	6.8	4.6- 9.8
Ngounie	June 2006	233	22	52,115	454	16	3.5	2.1- 5.8
Ogooue Ivindo	June 2007	197	41	34,540	621	13	2.1	1.2- 3.6
Ogooue Lolo	September 2007	190	18	36,877	422	7	1.7	0.7- 3.5
Ogooue Maritime	May 2008	188	10	12,940	206	1	0.5	0- 2.7

**TOTAL**		**2048**	**220**	**387,670**	**4342**	**269**	**6.2**	**5.5- 7**

**Table 2:** Prevalence of *Plasmodium falciparum *carriage, based on thick blood films, among adults, according to the ecosystem

*Ecosystems*	*Villages surveyed*	*n*	*+*	*%*	*95% IC*	p value
***Total population***	***220***	***4342***	***269***	***6.2***	***5.5- 7.0***	

Lakeland	24	439	11	2.5	1.3- 4.6	
Savannah	22	450	18	4	2.5- 6.4	0.0002
Forest	174	3453	240	7	6.1- 7.9	

*Grassland forest*	62	918	100	10.9	9- 13.1	
*Mountain forest*	22	399	14	3.5	1.9- 5.8	
*Interior forest*	50	1313	89	6.8	5.5- 8.3	<0.0001
*North-eastern forest*	40	823	37	4.5	3.2- 6.2	

## Discussion

This large survey of asymptomatic *P. falciparum *infection in 220 randomly selected Gabonese villages showed overall prevalence rates of 6.2% in adults and 5% in children. In previous surveys, *P. falciparum *carriage was found in 19 of 158 adults hospitalized in Libreville, the capital of Gabon [5], and in 12% of 493 adults tested in villages near Lambaréné, central Gabon [6]. In Uganda and Mozambique, the prevalence of *P. falciparum *infection in adults, based on direct examination, was reported to be respectively 29.1% and 14% [7,8].

This is the largest survey of *P. falciparum *infection among Gabonese adults based on the thick blood technique. The prevalence reached 37.2% in one village, the highest rate ever reported in this country. The thick blood technique is known to be less sensitive than PCR [6], indicating that the true prevalence is higher than observed here. The prevalence tended to be higher in forest villages. Surprisingly, however, only 5% of children living in forest villages appeared to be infected. This may due to the fact that the children were sampled in September, just before the rainy season (the period of intense transmission), although a marked decline in prevalence cannot be ruled out. Further studies are needed to confirm the decrease in the level of endemicity seen here, from some 65% thirty years ago [2,9]. The linear decrease observed with increasing age in the adult population is probably due to acquisition of immunity over time [10].

## Conclusion

Despite new control programmes, *P. falciparum *carriage remains frequent among Gabonese adults. More efforts are, therefore, needed especially in hard-to-access rural areas. Malaria screening of blood donors should be maintained, even though this mode of transmission is rare. Vector control is unrealistic in forest areas. Nevertheless, the use of insecticide-treated bed nets and treatment of individuals with asymptomatic infection or clinical malaria might reduce the transmission rate.

## Conflicts of interest

None to declare

## Authors' contributions

Conceived and designed the experiments: EML, DN. Performed the experiments: DN. Analysed the data: DN, JPA, JPG. Wrote the paper: DN. All authors read and approved the final manuscript.
